# Are Adolescents with Higher Openness More Creative Under Stress? The Mediating Role of Stress Perception and Cognitive Flexibility

**DOI:** 10.3390/jintelligence14020032

**Published:** 2026-02-14

**Authors:** Yifan Wang, Jialing Liu, Yadan Li, Haijun Duan

**Affiliations:** Key Laboratory of Modern Teaching Technology, Ministry of Education, Shaanxi Normal University, Xi’an 710062, China; wangyifan0929@snnu.edu.cn (Y.W.); liujialing2002@snnu.edu.cn (J.L.); liyadan@snnu.edu.cn (Y.L.)

**Keywords:** openness to experience, stress, creative tendency, stress perception, cognitive flexibility

## Abstract

Stress is a major risk factor for creativity development in adolescents. This study explored the protective effect of openness on creative tendency under stress and revealed the underlying mechanisms from the perspectives of stress perception and cognitive flexibility. A total of 1489 junior high school students (*Mage* = 13.65 years, *SD* = 0.74) participated in the study. The results showed that stress perception and cognitive flexibility sequentially mediated the negative effect of stressors on creative tendency, and openness moderated this process. Individuals with high openness had lower stress perception and higher cognitive flexibility at the same level of stressors, thus showing a higher creative tendency. However, the protective effect of openness diminished as the stress level increased. We concluded that openness could buffer the negative effects of stress on creative tendency to some extent. These findings highlight the importance of positive personality traits and provide a theoretical guide for cultivating creative qualities.

## 1. Introduction

Creativity is a fundamental psychological construct that supports innovation and adaptive problem-solving. In the context of rapid technological change and increasing societal complexity, creativity is widely regarded as an essential core competence for talent in the 21st century (OECD, 2008). The dynamic interplay between individual dispositions and situational factors shapes the trajectory of creative development (Sternberg, 2025). Early life stress is a critical environmental factor that may cause irreversible damage during the development of creativity in adolescence. A growing body of research evidence suggests that the induction of stress has damaging effects on creative processes (Duan et al., 2020; Y. Wang et al., 2022, 2023). Maintaining creative vitality in a stress-filled environment has become a new challenge. A tendency towards creative activities and processes is a key factor driving creative behaviors and outputs (P.-Z. Chen et al., 2020; L.-N. Li et al., 2022; Lopez-Persem et al., 2024; Shi et al., 2013; Williams, 1980). In this study, building upon an in-depth understanding of the underlying mechanisms, we examined whether openness—a core dimension of the Big Five personality model—could serve as a protective factor, helping adolescents maintain higher creative tendency following exposure to stressors.

### 1.1. The Effect of Stressors on Creative Tendency

Creativity is the ability to generate novel and appropriate ideas, solutions, or products, and it involves multiple cognitive processes such as divergent thinking and insight-based problem solving (Jiao et al., 2017; C. C. Wu et al., 1998; W. Shen et al., 2018; C.-L. Wu et al., 2020). Creative tendency is one of the critical components of creativity (L. Chen, 1999; Dai & Shen, 2008). It refers to a relatively stable set of positive attitudes and mental dispositions that orients individuals toward engagement in creative activities and processes (Williams, 1980). Stressor exposure can induce a series of non-specific physiological and psychological responses that help the organism to quickly conserve energy to cope with the stressors. Creativity is highly sensitive to stress responses induced by stressors (Beversdorf, 2019). Meta-analytic evidence indicates that stressors are generally negatively associated with creative performance (Byron et al., 2010). However, the effects of stressors on creative tendency, particularly in adolescents, remain largely underexplored. According to the cognitive appraisal theory of stress (Lazarus & Folkman, 1984), external stressors first give rise to perceived stress through individuals’ subjective appraisal of situational demands. This stress perception may directly diminish individuals’ approach tendencies toward creative engagement. Over time, cognitive impairments resulting from prolonged stress may further undermine creative tendency. Accordingly, perceived stress and cognitive impairment are proposed as two theoretically aligned mediators linking stressors to creative tendency.

#### 1.1.1. The Mediating Role of Stress Perception in the Effect of Stressors on Creative Tendency

From the perspective of stress processing, people will appraise an imbalance between the demands of a situation and their available coping resources, leading to individual variation in subjective stress perceptions (Glandorf et al., 2022). Compared to objective stressor exposure, subjective stress perception induced by stressors directly affects individuals’ behavioral and cognitive response patterns. It is commonly accompanied by negative emotional experiences such as fear, anxiety, and depression (Besser & Shackelford, 2007; Messineo & Tosto, 2023; X. Zhou & Huang, 2023; Gilar-Corbi et al., 2025). Negative emotions elicit a shift in motivational orientation, characterized by diminished approach motivation and heightened avoidance motivation, thereby promoting reliance on passive emotional coping strategies (Cheng et al., 2022; Davis, 2009; Ivcevic & Brackett, 2015; M. Wang et al., 2024). Such coping strategies may narrow attention scope and diminish internal associative activity, causing increased persistence and less flexibility (Alexander et al., 2007; Gabrys et al., 2019; Goldfarb et al., 2017; Guo et al., 2024). Therefore, at the same level of stress exposure, individuals with higher perceived stress may exhibit lower creative tendencies and performance. As evidence, a negative correlation between subject stress perception and creativity performance has been demonstrated (Atta et al., 2025; Danese & McEwen, 2012; Fiori et al., 2022; H. Yao et al., 2023). Consistently, research has shown that higher perceived stress is associated with lower creativity, partly through reduced subjective well-being and increased depressive symptoms (S. Zhou et al., 2025). Importantly, recent research among Chinese high school students indicates that perceived stress directly weakens the positive effect of self-esteem on creative tendency (Gao et al., 2024). Drawing on these arguments, we proposed the following:

**H1.**  *Stressors impair creative tendency.*

**H2.**  *Stressors weaken creative tendency by increasing stress perception.*

#### 1.1.2. The Mediating Role of Cognitive Flexibility in the Effect of Stressors on Creative Tendency

In creative processing, from a cognitive perspective, impaired cognitive flexibility may represent a key mechanism through which stressors undermine creativity and creative tendency. Studies in children and adolescents have shown a hindering effect of stressor exposure on the development of cognitive flexibility (Harms et al., 2018; Hostinar et al., 2012; Lewis-Morrarty et al., 2012; Spann et al., 2012). Cognitive flexibility, as a central variable influencing creative behavior, is positively associated with high creative achievement (Dreu et al., 2011; Dwertmann et al., 2025). High levels of early stressor exposure, through decreasing cognitive flexibility, further encourage a preference for habituated dominating behavior over innovative responses in the face of changed environments (Huang et al., 2024; X. Zhou et al., 2020).

Importantly, the impact of stressor exposure on cognitive flexibility may depend not only on objective stress levels but also on individuals’ subjective stress perception. Even under equivalent levels of stressor exposure, higher perceived stress is likely to elicit stronger negative emotional responses, which in turn more severely undermine cognitive flexibility. Consistent with this, perceived stress has been shown to function as a critical mediating variable in the association between stressors and cognitive flexibility (Kalia & Knauft, 2020; Knauft et al., 2021; Spann et al., 2012). However, whether stress perception and cognitive flexibility serve as key chain mediating pathways influencing adolescents’ creative tendency and whether personality traits can mitigate the adverse effects of stressors by altering these pathways remain to be further explored. Drawing on these arguments, we proposed the following:

**H3.**  *Stressors weaken creative tendency by reducing cognitive flexibility.*

**H4.**  *Stress perception and cognitive flexibility play a chain mediating role in the effect of stress on creative tendency.*

### 1.2. Moderate Role of Openness in the Effect of Stressors on Creative Tendency

#### 1.2.1. The Effect of Openness on Stressors and Stress Perception

Personality, a stable trait developed from childhood, influences cognition, thinking, and behavior. As a core dimension of the Five-Factor Model, openness to experience refers to individuals’ receptivity to and interest in novel experiences, ideas, and situations (Costa & McCrae, 1976). Openness is an important buffering trait in reducing stress perception (Zhu et al., 2022). Individuals with high openness showed lower heart rate and blood pressure changes under stress (Lü et al., 2016; Xin et al., 2017). They also showed lower cortisol and respiratory sinus arrhythmias after stress exposure (Bibbey et al., 2013; Lü et al., 2016). Moreover, evidence from acute stress paradigms demonstrates that individuals high in openness exhibit more adaptive cardiovascular and hemodynamic response trajectories across the entire stress exposure period, suggesting a buffering role of openness in physiological stress reactivity (Ó Súilleabháin et al., 2018). This supports the idea that openness can provide energetic motivation for faster coping and adaptation to stressors and thus may reduce individuals’ subjective stress perception.

In terms of psychological response differences, individuals with high openness are more inclined to make positive stress assessments of stressors. Specifically, individuals assess stressors based on whether their psychological and material resources are sufficient to cope with the demands of the situation. If psychological resources exceed the needs of the situation, the stressor is assessed as a ‘challenge’. Conversely, if psychological resources are insufficient to cope with the needs of the situation, the stressor is assessed as a ‘threat’ (Seery, 2011). Different outcomes of the assessment can affect subjective stress perceptions (e.g., negative emotions, anxiety, and tension) and can have different impacts on creative tendency. Most research suggests that individuals with high openness have higher assessments of self-efficacy and perceive lower task demand and task difficulty, as well as greater control over tasks (Bibbey et al., 2013; Mornar et al., 2022; Penley & Tomaka, 2002; Sanatkar & Rubin, 2020). As a result, they typically have higher psychological resources than task demands and are more likely to identify stressors as ‘challenges’ that are favorable to their growth. Challenging stressors increase motivation levels, leading them to increase their efforts to cope with the demands of the stress and to adopt a problem-oriented coping style (Agbaria & Mokh, 2022). To minimize the perceived distress generated by stressors, challenging assessments, and positive coping styles motivate individuals to proactively generate more problem solutions with more flexible thinking (Anderson et al., 2004; Carver, 2004). Moreover, this adaptive coping style could motivate them to regulate negative emotions through positive emotion regulation strategies during problem solving under stress (Barańczuk, 2019; Conner & Silvia, 2015; Penley & Tomaka, 2002). Positive emotional states can diminish psychological stress perception, which in turn promotes creative tendency by activating approach motivation and increasing cognitive flexibility (Ivcevic & Brackett, 2015). Studies have shown that highly open adolescents can mitigate the impact of stress from cyberbullying through cognitive flexibility and social support, whereas those with low openness are more susceptible to such stress and tend to fall into negative psychological cycles (Y. Wang et al., 2023). Drawing on these arguments, we proposed the following:

**H5a.**  *Openness plays a moderating role in the relationship between stressors and creativity.*

**H5b.**  *Openness can diminish the negative effect of stressors on creativity by reducing stress perception.*

#### 1.2.2. The Effect of Openness on Cognitive Flexibility and Creative Tendency

Creative qualities such as curiosity, insight, and imagination have been recognized as core characteristics of open-mindedness, which makes openness the best predictor of creativity (DeYoung et al., 2012; Karwowski & Lebuda, 2016; Theurer et al., 2021). A strong correlation between them has been supported by multiple pieces of evidence across measurement tasks and age (Samimi Dehkordi et al., 2025; Beaty et al., 2018a; Puryear et al., 2017; Lin & Vartanian, 2018). Nevertheless, our understanding is limited regarding whether the promotion of creative tendency via openness can still be expected to occur in specific threatening situations.

Openness to more and more experiences motivates individuals to continuously form novel connections, developing creativity-related cognitive control processes such as cognitive flexibility (Baas et al., 2013; X. Chen et al., 2022). Longitudinal evidence shows that openness to experience predicts changes in adolescents’ divergent thinking, supporting the role of openness in the development of creativity during adolescence (Asquith et al., 2024). As evidence, in one study, a high-openness group showed better semantic memory network connectivity and flexibility (Samuel et al., 2023; Christensen et al., 2018). Given differences in brain structure and function, individuals with high openness were able to achieve dynamic and efficient cooperation with various brain networks during the creative cognitive process, which regulated goal-orientated behavior and cognitive flexibility to actively guide the processes of idea generation and evaluation (Beaty et al., 2016, 2018a; Lin & Vartanian, 2018; W. Li et al., 2015). The cortical volume and activation of brain regions involved in inhibitory or warning responses were also negatively correlated with the openness trait, reflecting a tendency to attenuate inhibitory responses (Kapogiannis et al., 2013; M. Li et al., 2024). Openness also contributes in part to cognitive flexibility during creative processing under stress. As a consequence, the neural resources used to maintain creativity levels in individuals with high openness may reorganize rapidly during stressful states, helping to re-establish a new homeostasis with higher stability. Drawing on these arguments, we proposed the following:

**H5c.**  *Openness can diminish the negative effect of stressors on creativity through increasing cognitive flexibility.*

### 1.3. The Present Study

**Figure 1:**
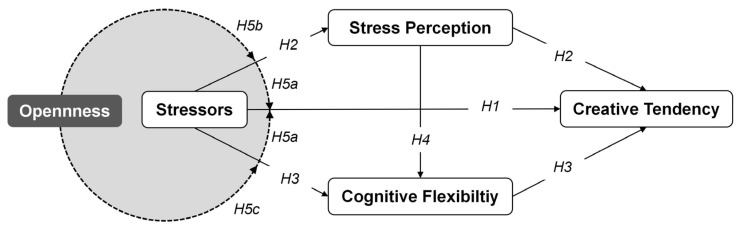
Theoretical model of this study.

Drawing on cognitive appraisal theory of stress, cognitive models of creativity emphasizing flexibility, and personality-based resilience perspectives, the present study proposes an integrated theoretical framework to explain how stressors influence adolescents’ creative tendency through emotional-motivational and cognitive control pathways, and how openness functions as a protective factor in this process. The chain mediating role of stress perception and cognitive flexibility in the influence of stressors on creative tendency is first verified. Based on this, the moderating effects of openness are further explored to verify whether it could form a protective ring to block the negative effects of stress on adolescents’ creative tendency through the moderation of the mediating variables. The proposed research model is illustrated in Figure 1.

## 2. Materials and Methods

### 2.1. Participants

A total of 1943 junior high school students (*Mage* = 13.25 years; *SD* = 0.97; range: 13–16 years) participated in the study. Students who had physical or mental health conditions that made it difficult for them to complete the questionnaire were excluded from the study. Questionnaires were administered in the form of a field test, with the answers collected on the computer. Each questionnaire was preceded by complete instructions and was set up so that all questions could be answered before submission. To further investigate the predictive effect of stressors on adolescent development, data were collected at two time points. At Time 1 (March 2024), stressor exposure and openness to experience were assessed. After a six-month interval, at Time 2 (September 2024), perceived stress, cognitive flexibility, and creative tendency were measured. Due to some participants not completing the assessment at Time 2, a total of 1489 students (*Mage* = 13.65 years; *SD* = 0.74; range: 13–16 years) were included in the final data analysis. Among them, 717 (48.2%) were male, and 772 (51.8%) were female. A total of 426 (28.6%) were in the first year, 630 (42.3%) in the second year, and 433 (29.1%) in the third year. Participants were drawn from across Shaanxi Province, China, including 46 from provincial capitals (3.1%), 200 from prefecture-level cities (13.4%), 813 from counties (54.6%), and 430 from townships (28.9%). Participants and their parents were informed of the research purpose and data confidentiality procedures. The study adhered to ethical guidelines, and we obtained approval from the institutional review board.

### 2.2. Measures

#### 2.2.1. Stressors

Stressors were measured using the Stressors Scale for Secondary School Students (Zheng & Chen, 1999). The scale consisted of a 39-item stressor scale, which included seven dimensions of stressors from schoolwork, teachers, the family environment, parenting style, peers, social culture, and mind–body factors. Example items include feeling tense during exam preparation, experiencing sleep problems, and having quarrels or conflicts with classmates or friends. Participants recalled whether the items described in the scale had occurred in the past and the extent to which the occurrence of these events had affected them. A 5-point scale was used, with 0 being no occurrence or no effect and 4 being extremely severe. Higher scores indicate greater stressor intensity. The scale reflects typical stressful life events experienced by secondary school students. The overall internal consistency coefficient of the scale was reliable in this study (Cronbach’s alpha = 0.970).

#### 2.2.2. Creative Tendency

Creative tendency was measured using the Creative Tendency Questionnaire for Adolescents (J. L. Shen et al., 2005). The questionnaire consisted of 37 items in five dimensions: self-confidence, curiosity, exploration, challenge, and persistence. The items were scored on a 5-point scale. Higher scores indicated more creative behavior. In this study, the overall internal consistency coefficient of the scale was reliable (Cronbach’s α = 0.887).

#### 2.2.3. Openness in Personality

Openness in personality was measured using the Neuroticism Extraversion Openness Five-Factor Inventory (NEO-FFI) (Costa & McCrae, 1992). Although the full Big Five personality inventory was administered, the present study focused exclusively on openness to experience, as it was theoretically most relevant to the research questions, and other personality dimensions were therefore not included in the analyses. The openness dimension of the NEO-FFI consists of 12 items. The scale was rated on a 5-point scale, with 5 being “strongly agree” and 1 being “strongly disagree”. The Chinese version of the NEO-FFI has shown satisfactory reliability and validity in previous studies and is commonly used in research with Chinese participants (R. S. Yao & Liang, 2010; Y. Zhou et al., 2019) The strong internal consistency reliability (Cronbach’s α = 0.899) suggested that the scale was reliable.

#### 2.2.4. Perceived Stress

The subjectively perceived stress was measured using the 14-item Perceived Stress Scale, which was translated and adapted from the English Perceived Stress Scale (Cohen et al., 1983). A 5-point Likert scale was adopted in the questionnaire, with 5 being “almost always” and 1 being “almost never”. Participants were asked to rate their perceived level of stress over the past six months. In this study, the strong internal consistency reliability (Cronbach’s α = 0.804) suggested that the scale was reliable.

#### 2.2.5. Cognitive Flexibility

Cognitive flexibility was measured using the Cognitive Flexibility Scale (Martin & Rubin, 1995). This scale mainly measures individuals’ perceived ability to identify multiple options and flexibly adapt to situational demands, and it has been widely used in studies involving adolescent populations. It includes 12 items that are rated on a 6-point Likert scale, with 6 being “strongly agree” and 1 being “strongly disagree”. In this study, the reliability was acceptable (Cronbach’s α = 0.794).

### 2.3. Data Analysis

Data analyses were conducted using SPSS 26.0 and AMOS 23.0. Descriptive statistics and Pearson correlations were computed to examine associations among variables. Confirmatory factor analysis (CFA) was performed to test discriminant validity among the five constructs using structural equation modeling. To reduce model complexity, item parceling was applied, with three parcels created for each latent variable using the balancing method. Model fit was evaluated using ***χ*^2^/*df***, **CFI**, **TLI**, **RMSEA**, and **SRMR**.

Mediation and chain mediation effects were tested using the PROCESS macro (Model 6) with 5000 bootstrap samples to estimate bias-corrected 95% confidence intervals. Moderation and moderated mediation effects of openness were examined using PROCESS Models 1 and 7. Gender and age were included as control variables in all regression-based analyses.

## 3. Results

### 3.1. Confirmatory Factor Analysis

Confirmatory factor analysis was conducted to examine the discriminant validity among creative tendency, stressors, openness personality, stress perception, and cognitive flexibility. During the process of structural equation modeling, the measurement scales included a large number of items, which might lead to substantial parameter estimation bias. To improve the validity of the model fit indices, the item parceling approach was adopted to reduce the number of indicators for each latent variable (Y. Wu & Wen, 2011). As the present study was focused on the discriminability among the variables rather than the content-level discriminant validity within each construct, the balancing method was used for parceling. Each scale was divided into three parcels with approximately equal factor loadings and variances to minimize within-construct heterogeneity.

As shown in Table 1, among the five competing models, the five-factor model demonstrated the best model fit, with **χ^2^/*df*** = 4.917, **CFI** = 0.965, **TLI** = 0.975, **RMSEA** = 0.051, and **SRMR** = 0.014. These results indicate that the variables examined in this study exhibited good discriminant validity.

### 3.2. Common Method Bias Test

Since all the questionnaires used in this study were based on self-reported measures, there was a potential risk of common method bias. To minimize this concern, Harman’s single-factor test was conducted to statistically examine the extent of common method variance (Harman, 1976; Malhotra et al., 2006). All measurement items from the study were entered into an exploratory factor analysis. The results showed that 18 factors with eigenvalues greater than 1 were extracted, and the first factor accounted for 21.45% of the total variance, which is below the critical threshold of 40%. The results indicated that common method bias was minimal.

### 3.3. Correlation Analysis

Pearson correlation analysis revealed strong correlations among all five key variables (Table 2). Gender was coded as 1 = male and 2 = female. Given the significant correlation between gender and age with the other variables, they were included as control variables in the subsequent analysis.

### 3.4. Mediation Analysis

**Figure 2:**
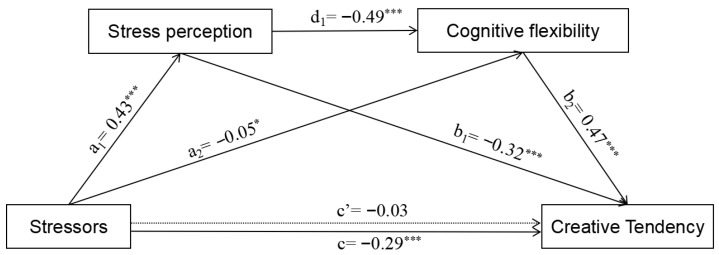
Chain-mediated path diagram of stressors on creative tendency. * *p* < 0.05, *** *p* < 0.001.

The negative effect of stressors on creative tendency was significant (*β* = −0.29, *p* < 0.001), supporting Hypothesis 1. The results of the chain mediation model are shown in Figure 2. Stressful events positively predicted stress perception (*a*_1_ = 0.43, *p* < 0.001), while stress perception negatively predicted creative tendency (*b*_1_ = −0.32, *p* < 0.001, 95% CI = [−0.1627, −0.1095]), supporting Hypothesis 2. Stress negatively predicted cognitive flexibility (*a*_2_ = −0.05, *p* = 0.027), while cognitive flexibility positively predicted creative tendency (*b*_2_ = 0.47, *p* < 0.001, 95% CI = [−0.0502, −0.0005]), supporting Hypothesis 3. In addition, the negative predictive effect of stress perception on cognitive flexibility was significant (*d*_1_ = −0.49, *p* < 0.001). This further showed that the chain mediation effect took up 60.73% of the total effect, while the 95% CIs were [−0.1174, −0.0802]. Hypothesis 4 was supported.

### 3.5. Testing for the Moderated Mediation Model

**Figure 3:**
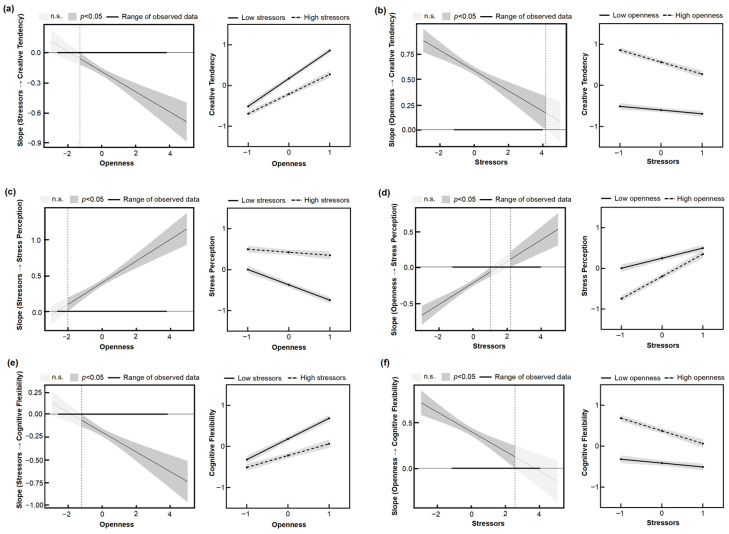
Plots illustrating the moderation effect and the Johnson–Neyman regions. Changes in slope of stressors with creative tendency (**a**), stress perception (**c**), and cognitive flexibility (**e**) as dependent variables, respectively. Changes in slope of openness with creative tendency (**b**), stress perception (**d**), and cognitive flexibility (**f**) as dependent variables, respectively.

To test Hypothesis 5, a series of moderation and moderated mediation analyses were conducted to examine whether openness moderated the direct and indirect effects of stressors on creative tendency. The moderation model (PROCESS Model 1) was first employed to examine the moderating role of openness in the effect of stressors on creative tendency. The results indicated that openness significantly positively predicted creative tendency (*β* = 0.58, *p* < 0.001), and the interaction between stressors and openness also significantly predicted creative tendency (*β* = −0.10, *p* < 0.001), supporting Hypothesis 5a. Openness was probed at one standard deviation above and below the mean. As shown in Figure 3a, stressors negatively predicted creative tendency in the high- (simple slope = 0.09, *p* = 0.001), moderate- (simple slope = −0.19, *p* < 0.001), and low-openness groups (simple slope = −0.29, *p* < 0.001). However, individuals with high openness exhibited greater creative tendency than those with low openness, regardless of the level of stressors, suggesting that openness enhances creative tendency under stress. Johnson–Neyman simple slope analysis indicated that as openness scores increased, the negative predictive effect of stressors on creative tendency became stronger, suggesting that creative tendency in individuals with high openness is more sensitive to stressors.

To further investigate the protective effect of openness on creative tendency at different levels of stress, a moderation analysis was conducted with stressors as the moderator of the effect of openness on creative tendency (Figure 3b). The interaction between stressors and openness significantly predicted creative tendency (*β* = −0.10, *p* < 0.001). Openness positively predicted creative tendency when stressors were low (simple slope = 0.68, *p* < 0.001), moderate (simple slope = 0.58, *p* < 0.001), or high (simple slope = 0.48, *p* = 0.001). Johnson–Neyman analysis revealed that the positive predictive effect of openness on creative tendency gradually weakened as stressors increased. These results further demonstrate that openness can buffer the negative impact of stress on creative tendency to some extent. However, this protective effect diminishes as stress levels decrease.

**Figure 4:**
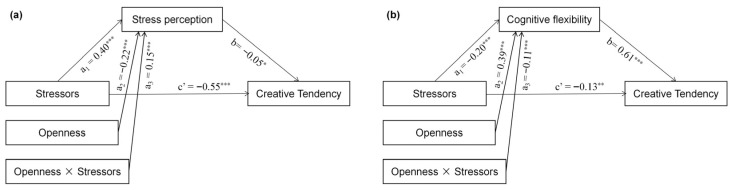
Results of the moderated mediation model. (**a**) Stress perception as the mediator and openness as the moderator. (**b**) Cognitive flexibility as the mediator and openness as the moderator. * *p* < 0.05, ** *p* < 0.01, *** *p* < 0.001.

PROCESS Model 7 was employed to examine whether openness could attenuate the effect of stressors on adolescents’ creative tendency by reducing perceived stress (Figure 4a). The results indicated that openness significantly negatively predicted perceived stress (*β* = −0.22, *p* < 0.001), and the interaction between stressors and openness also significantly predicted perceived stress (*β* = 0.15, *p* < 0.001). As shown in Figure 3c, stressors positively predicted perceived stress in both the high- (simple slope = 0.54, *p* < 0.001), moderate- (simple slope = 0.40, *p* < 0.001), and low-openness group (simple slope = 0.24, *p* < 0.001). However, individuals with high openness reported lower perceived stress than those with low openness, regardless of the level of stressors, demonstrating that openness can reduce perceived stress. Furthermore, bootstrap analysis with 5000 resamples revealed a significant moderated mediation effect (95% CI = [−0.1149, −0.0507]), supporting Hypothesis 5a. These results suggest that openness can significantly reduce perceived stress under stress, thereby enhancing creative tendency.

To further examine under which levels of stressors the negative effect of openness on perceived stress is effective, the moderating effect of stressors in the relationship between openness and perceived stress was further tested (Figure 3d), with openness as the independent variable, perceived stress as the dependent variable, and stressors as the moderator. The interaction between stressors and openness significantly predicted perceived stress (*β* = 0.15, *p* < 0.001). Openness was assessed at one standard deviation above and below the mean. The negative predictive effect of openness on perceived stress remained significant when stressors were low (simple slope = −0.37, *p* < 0.001), moderate (simple slope = −0.08, *p* < 0.001), and high (simple slope = −0.07, *p* = 0.02). Johnson–Neyman analysis indicated that, as stressors increased, the slope of the negative effect of openness on perceived stress gradually became smaller, indicating a weakening of the effect. Bootstrap analysis with 5000 resamples again confirmed a significant moderated mediation effect (95% CI = [−0.0889, −0.0373]). These findings indicate that openness can attenuate the negative impact of stress on creative tendency by reducing perceived stress. However, this protective effect diminishes as stress levels decrease.

PROCESS Model 7 was further employed to examine whether openness could mitigate the effect of stressors on adolescents’ creative tendency by enhancing cognitive flexibility. The results indicated that openness significantly positively predicted cognitive flexibility (*β* = 0.39, *p* < 0.001), and the interaction between stressors and openness also significantly predicted perceived stress (*β* = −0.11, *p* < 0.001). As illustrated in the interaction plot in Figure 3e, individuals with high openness exhibited greater cognitive flexibility than those with low openness, regardless of stressor levels, demonstrating that openness can enhance cognitive flexibility. Bootstrap analysis with 5000 resamples revealed a significant moderated mediation effect (95% CI = [−0.1103, −0.0197]), supporting Hypothesis 5b (Figure 4b). These findings suggest that under stress, higher openness is associated with greater cognitive flexibility, which in turn protects creative tendency from being impaired.

To further examine the conditions under which openness effectively enhances cognitive flexibility, the moderating effect of stressors in the relationship between openness and cognitive flexibility was further tested (Figure 3f), with openness as the independent variable, cognitive flexibility as the dependent variable, and stressors as the moderator. The interaction between stressors and openness significantly predicted perceived stress (*β* = −0.17, *p* < 0.001). Probing at one standard deviation above and below the mean revealed that the positive predictive effect of openness on cognitive flexibility remained significant when stressors were high (simple slope = 0.29, *p* < 0.001), moderate (simple slope = 0.39, *p* < 0.001), and low (simple slope = 0.50, *p* < 0.001). Johnson–Neyman analysis indicated that as stressors increased, the slope of the positive effect of openness on cognitive flexibility gradually decreased, reflecting a weakening of the effect. Bootstrap analysis with 5000 resamples further confirmed a significant moderated mediation effect (95% CI = [−0.0833, −0.0148]). Taken together, these results indicate that, under equivalent levels of stress, individuals with higher openness exhibit lower perceived stress and greater cognitive flexibility, which in turn buffer the detrimental effects of stressors on creative tendency, thereby supporting Hypothesis 5.

### 3.6. Full Structural Model Analysis

**Figure 5:**
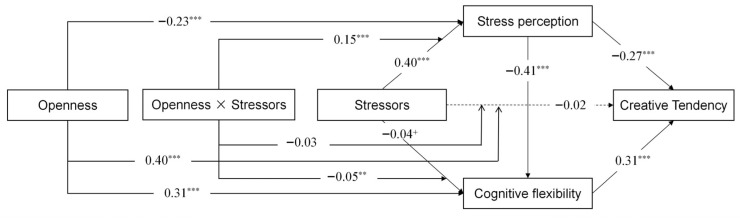
Testing for five-factor measurement model. ^+^ *p* < 0.07, ** *p* < 0.01, *** *p* < 0.001.

According to the hypothesis of the study, path analyses were performed to test the full model with five variables (Figure 5). The results showed that the fit statistics met the following criteria: ***χ*^2^/*df*** = 3.932, **CFI** = 0.998, **TLI** = 0.983, **RMSEA** = 0.044, **SRMR** = 0.016. To further examine whether creative thinking could influence actual creative behavior, data from the Runco Ideational Behavior Scale were collected and incorporated into the model. The scale consists of 23 items rated on a 5-point Likert scale and is primarily used to assess creative behaviors related to the generation of original and divergent ideas (Runco et al., 2001). As seen in Figure S1, the structural model including creative behavior showed a satisfactory model fit. The results demonstrated that creative tendency further influenced creative behavior, while openness played a significant moderating role in the effects of stressors on both creative tendency and creative behavior.

## 4. Discussion

The purpose of this study was to investigate the moderating role of openness in the influence of stress on creative tendency in adolescents. The findings showed that stressors could negatively affect creative tendency through the mediating effects of stress perception and cognitive flexibility. Openness can be a protective factor against the impairment of creative tendency by reducing stress perception and increasing cognitive flexibility. However, in individuals under stress, the protective effect of high openness diminishes with increasing stress levels.

### 4.1. The Negative Effect of Stressors on Creative Tendency

Allostatic load or overload caused by stressors can negatively impact the neuroendocrine system and brain function, both of which are crucial in the development of cognitive abilities (Eiland & Romeo, 2013). Adolescence is a critical period for physical and psychological development, making individuals more susceptible to stressful situations. This study examined the impact of early stressor exposure on adolescents’ creative development, revealing significant negative predictive effects. In light of this, the results further support the chain-mediated roles of stress perception and cognitive flexibility in the impact of stressors on creative tendencies.

When confronted with the same stressors, individuals vary in their subjective perception based on their assessment of available resources (Danese & McEwen, 2012). This variation in subjective perception determines the actual impact of stressors on creativity. At the cognitive level, we found that both stressors and stress perception could negatively influence creative tendency by impairing cognitive flexibility. According to the dual pathway to creativity model, creative cognitive processing relies on the interplay between cognitive flexibility and cognitive persistence (De Dreu et al., 2008). Previous studies have demonstrated that high-intensity stress disrupts the balance between prefrontal and striatal dopamine, leading to low cognitive flexibility in individuals (Cools & D’Esposito, 2011). Having too much persistence and too little flexibility inhibits individuals from exploring extensively in the problem space, which affects the tendency to behave creatively (Boot et al., 2017). As students progress through grades, this cognitive impairment increases the likelihood of developing learning difficulties. Therefore, it is crucial to provide timely attention and interventions for adolescents experiencing excessive stress to prevent it from having detrimental effects on cognitive development and academic achievement.

### 4.2. Openness Buffered the Negative Impact of Stressors on Creative Tendency

Individual traits influence the direction and stability of the impact of stress on creativity. For instance, Duan et al. (2019) found that individuals with low trait anxiety were better able to inhibit irrelevant information under stress compared to those with high trait anxiety, thereby enhancing their creativity performance. Understanding the moderating role of individual differences can help foster adolescents’ potential and strengths. This study demonstrated that openness is a valuable psychological resource for problem-solving and may act as a buffer against stress.

Consistent with previous studies, this research confirmed the mitigating effect of openness on stress, as individuals with higher openness exhibited lower stress perception. At the behavior level, individuals with high openness typically adopt “problem-focused” adaptive emotion regulation strategies in stressful situations, which contribute to more positive emotions and greater emotional regulation abilities (Barańczuk, 2019). Additionally, they tend to perceive higher self-efficacy, lower task difficulty, and reduced threat (Penley & Tomaka, 2002). A positive appraisal of stressful situations, combined with more effective emotional regulation, can lower subjective stress perception. This diminished stress perception serves as a fundamental protective factor for the development of creativity.

In addition to reduced stress perceptions, we found that individuals with high openness maintained greater cognitive flexibility, which helped mitigate the negative impact of stress on creative tendencies. Individuals with high openness are characterized by imagination, curiosity, flexibility, and high intelligence, all of which are closely associated with strong creative tendencies. The personality trait of openness is linked to brain regions involved in dopaminergic pathways (Passamonti et al., 2015). Dopamine release in the brain activates the reward system, which enhances motivation for problem-solving and encourages individuals to engage in more exploratory behaviors (DeYoung et al., 2012). Dopamine release can also attenuate inhibitory functions while updating working memory representations, allowing for greater flexibility in attention direction and information processing in unpredictable environments (Cools & D’Esposito, 2011). Therefore, the high flexibility exhibited by individuals with high openness in stressful situations can provide some protection for the development of creative tendencies.

It is important to note that the moderating effects test revealed that high openness was linked to greater vulnerability to stress under high levels of stressors. One possible explanation is that individuals with low openness may adopt coping styles of avoidance or abandonment, which can somewhat mitigate the negative impacts of stressors. Conversely, the trait of high openness allows a wider range of external conflicts and threatening stimuli to enter cognitive processing under stress, increasing the risk of developing conditions such as schizophrenia or depression (Chiappelli et al., 2021). According to the U-shaped curve of dopamine activity, individuals with higher inherent openness tend to have stronger baseline dopamine levels, making them more prone to exceed optimal arousal levels in highly stressful situations. This excessive activation of the neural arousal system may further disrupt the dynamic balance of brain functional networks (Shine et al., 2018; Beaty et al., 2015, 2018b; Q. Chen et al., 2025). Under high stress, this dynamic balance is particularly vulnerable, which can weaken the effective regulation of cognitive processing via executive control. For individuals high in openness, whose thinking is naturally more exploratory, impaired executive control under high stress may lead to overextended cognitive activity and imbalanced allocation of attention. This can result in excessive divergent thinking without convergence, leading to numerous exploratory behaviors that do not yield useful creative ideas, thereby increasing stress load and reducing the protective effect of openness. Käckenmester et al. (2019) found that blocking dopamine release improved performance in divergent thinking in individuals with high openness. Moreover, individuals with high openness are more willing to invest cognitive resources in processing complex and abstract information, engaging in deeper elaboration and organization of such information (DeYoung et al., 2005). However, under prolonged high stress, their cognitive resources may become insufficient to support effective problem-solving, leading to rapid depletion of self-regulatory resources and resulting in fatigue and emotional exhaustion (Raio et al., 2013). Without sufficient recovery, this depletion may further reduce executive function and emotional regulation ability in highly open individuals, thereby weakening the protective role of openness. Future research may focus on approaches such as rest and mindfulness training to enhance cognitive and emotional regulatory resources in highly open individuals, thereby preventing exhaustion and dysfunction caused by chronic stress.

Furthermore, the relationships among perceived stress, personality traits, and creative tendency may be influenced by cultural background. In the Chinese cultural context, which is characterized by a strong collectivist orientation, adolescents grow up under academic and social expectation pressures, which may affect their understanding of stress and the expression of creative tendencies. Previous studies have also shown that the relationship between openness and creativity can be influenced by multicultural environments (Abu Raya et al., 2023; X. Zhou et al., 2023). Therefore, future research should consider cultural background to clarify the applicability of the findings.

### 4.3. Limitations and Future Directions

There are several limitations to the present study. First, this research primarily focused on measuring daily creative tendencies. Future studies could expand the dimensions of creativity to further validate the robustness of the results. Second, future research should increase the sample size and incorporate longitudinal methods to explore the sustained protective effects of openness in the next developmental periods. Moreover, since the sample in this study was drawn from Chinese adolescents, the generalizability of the findings to other cultural or educational contexts may be limited. Future studies should examine whether the protective role of openness in the relationship between stress and creative tendency represents a cross-culturally stable mechanism or varies due to cultural socialization. Finally, openness interacts with other positive and negative personal factors, such as optimism, resilience, and trait anxiety. In addition, the effects of gender and age were also observed. To better understand the complex mechanisms underlying the effect of stress on creativity, future research should consider a variety of cognitive, emotional, and gender-related factors.

## 5. Conclusions

In conclusion, this study first confirmed the protective effect of openness against creativity impairment induced by stressor exposure. It also revealed the underlying mediating mechanisms of stress perception and cognitive flexibility, providing a comprehensive explanatory framework for the influence of openness. These findings encourage educators to pay close attention to the current stressors faced by youth and to prioritize the cultivation of positive psychological qualities. Furthermore, the importance of creating a supportive environment and providing sustainable psychological resources for fostering creativity in adolescents is emphasized.

## Figures and Tables

**Table 1:** Fit test for measurement model comparison.

Models	*χ* ^2^	*df*	*χ* ^2^ */df*	CFI	TLI	RMSEA	SRMR
5-Factor Measurement Model	393.340	80	4.917	0.965	0.975	0.051	0.014
4-Factor Model ^a^	2601.413	84	30.969	0.790	0.812	0.142	0.069
3-Factor Model ^b^	2916.287	87	33.521	0.769	0.796	0.148	0.070
2-Factor Model ^c^	6610.697	89	74.277	0.620	0.540	0.222	0.074
1-Factor Model ^d^	12,117.472	90	134.639	0.543	0.161	0.300	0.111

*Notes:* Model ^a^: Stress perception and cognitive flexibility are combined into one factor. Model ^b^: Stressors and openness are combined into one factor. Stress perception and cognitive flexibility are combined into one factor. Model ^c^: Creative tendency and stressors are combined into one factor. Openness, stress perception, and cognitive flexibility are combined into one factor. Model ^d^: creative tendency, stressors combined, openness, stress perception, and cognitive flexibility are combined into one factor.

**Table 2:** Descriptive statistics and correlation matrix.

Variables	1	2	3	4	5	6	7
1. Gender	–						
2. Age	−0.049	–					
3. Stressors	0.049	0.028	–				
4. Creative tendency	−0.082 **	−0.083 **	−0.292 ***	–			
5. Openness	0.024	−0.050	−0.176 ***	0.627 ***	–		
6. Stress perception	0.132 **	0.088 **	0.434 ***	−0.574 ***	−0.309 ***	–	
7. Cognitive flexibility	−0.079 **	−0.125 **	−0.270 ***	0.642 ***	0.441 ***	−0.523 ***	–
*Mean*	1.52	13.65	34.93	120.35	39.42	38.95	44.23
*SD*	0.50	0.74	29.65	16.54	5.17	7.56	5.80

*Note:* ** *p* < 0.01, *** *p* < 0.001.

## Data Availability

Due to privacy restrictions, data are not publicly available but can be provided by the corresponding author upon reasonable request.

## Supplementary Materials

The following supporting information can be downloaded at: https://www.mdpi.com/article/10.3390/jintelligence14020032/s1. Figure S1: The full structural model including creative behavior.
