# Supplementary material for: Are Adolescents with Higher Openness More Creative Under Stress? The Mediating Role of Stress Perception and Cognitive Flexibility
Source: J Intell. 2026 Feb 14;14(2):32. doi: 10.3390/jintelligence14020032 (PMC12942368; doi:10.3390/jintelligence14020032)
Supplement: Supplementary file 1 [file jintelligence-14-00032-s001.zip › jintelligence-3970277-supplementary.pdf]

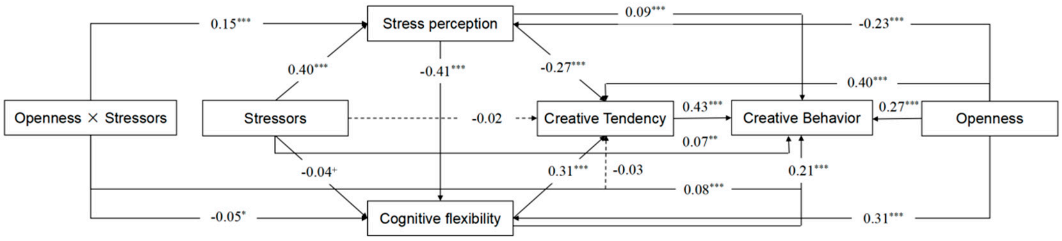

Figure S1 The full structural model including creative behavior. The full structural model showed satisfactory model fit: $\chi^2/df = 2.513$, CFI = 1.000, TLI = 0.991, RMSEA = 0.032, SRMR = 0.014. The results demonstrated that creative tendency further influenced creative behavior, while openness played a significant moderating role in the effects of stressors on both creative tendency and creative behavior. $^{+}p < 0.07$, $^{*}p < 0.05$, $^{**}p < 0.01$, $^{***}p < 0.001$.
